# A real-time stable-control gait switching strategy for lower-limb rehabilitation exoskeleton

**DOI:** 10.1371/journal.pone.0238247

**Published:** 2020-08-27

**Authors:** Ziming Guo, Can Wang, Chunning Song

**Affiliations:** 1 College of Electrical Engineering, Guangxi University, Nanning Guangxi, China; 2 CAS Key Laboratory of Human-Machine Intelligence-Synergy Systems, Shenzhen Institutes of Advanced Technology, Shenzhen, China; 3 Guangdong Provincial Key Laboratory of Robotics and Intelligent System, Shenzhen Institutes of Advanced Technology, Chinese Academy of Sciences, Shenzhen, China; National Huaqiao University, CHINA

## Abstract

Switching different gait according to different movements is an important direction in the study of exoskeleton robot. Identifying the movement intention of the wearer to control the gait planning of the exoskeleton robot can effectively improve the man-machine interaction experience after the exoskeleton. This paper uses a support vector machine (SVM) to realize wearer’s motion posture recognition by collecting sEMG signals on the human surface. The moving gait of the exoskeleton is planned according to the recognition results, and the decoding intention signal controls gait switching. Meanwhile, the stability of the planned gait during the movement was analyzed. Experimental results show that the sEMG signal decoding human motion intentional, and control exoskeleton robot gait switching has good accuracy and real-time performance. It helps patients to complete rehabilitation training more safely and quickly.

## Introduction

Currently, the occurrence and recurrence of strokes have been increasing. There are approximately 6.5 million stroke survivors in the U.S. [1]. Nearly 80% of stroke survivors experience significant impairments that require rehabilitation. Assistant equipment can help human legs and move synchronously with the human gait. Based on the principle of bionics, the lower limb exoskeleton robot provides support and assist function to the wearer through the external fixed support and driving mechanism of the human body. It can effectively address the need for assistance and rehabilitation training, and also reduce the shortage of professional nursing staff. So researchers programmed assistive devices to generate gait tracks [2] to help patients to undergo rehabilitation training. In this study, we intend to develop a method of controlling stable exoskeleton motion gait [3, 4] by surface muscle electrical signals to identify human motion intentions.

Berkeley biomimetics employs sensitivity amplification (SAC) control methods [5] to aid exoskeletons with HULC [6], which change the motion state of exoskeletons through very small interaction forces. Raytheon Company has developed XOS [7] exoskeleton robots. Cyberdyne in Japan, for its part, has developed a rehabilitation exoskeleton robot, HAL, for medical applications [8]. HAL captures surface electromyography (sEMG), plantar pressure signals, gyroscopes, and accelerometer signals using fuzzy control for information fusion of multimode sensors. ReWalk [9, 10] exoskeleton robots, Harvard University [11], MIT [12] from Elger Medical Technology in Israel have also made good progress. The University of Science and Technology of China has developed exoskeleton robots driven by servo motors [13, 14]. Zhejiang University has developed exoskeleton robots driven using pneumatic means [15]. University of Electronic Science and Technology have developed a lower limb exoskeleton robot controlled by fuzzy algorithms [16, 17]. The SIAT rehabilitation exoskeleton robot was studied by the Shenzhen Advanced Technology Research Institute of the Chinese Academy of Sciences. At present, it has been successfully achieved that disabled people drive autonomous standing and walking through servo motor [18–20]. Harbin engineering university has developed a walking exoskeleton robot [21] driven by a dc motor. Most exoskeleton developers use pre-programmed control modes and inertial measurement sensors to monitor user posture.

**Fig 1:**
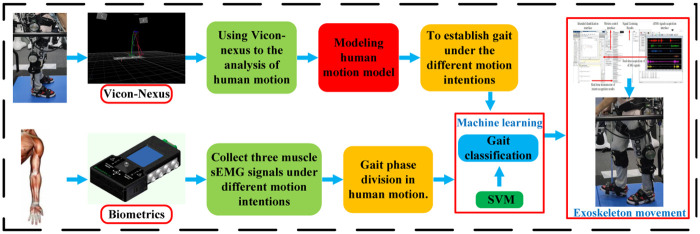
The main flowchart.

In general, the pre-programmed walking gait is passively controlled. The studies aforementioned are all based on pre-programmed control and rarely take into account the wearer’s human-computer interaction experience. To realize the interaction control between wearer and exoskeleton, this paper obtains human motion intention through support vector machine (Support Vector Machine, SVM) training arm sEMG signals [22–27]; establishes exoskeleton motion model and movement gait by real-time prediction of human motion intention, dividing gait phase; and constructs motion gait logic of exoskeleton device by real-time sEMG signal recognition. The overall structure is illustrated in Fig 1.

## Experimental equipment

**Fig 2:**
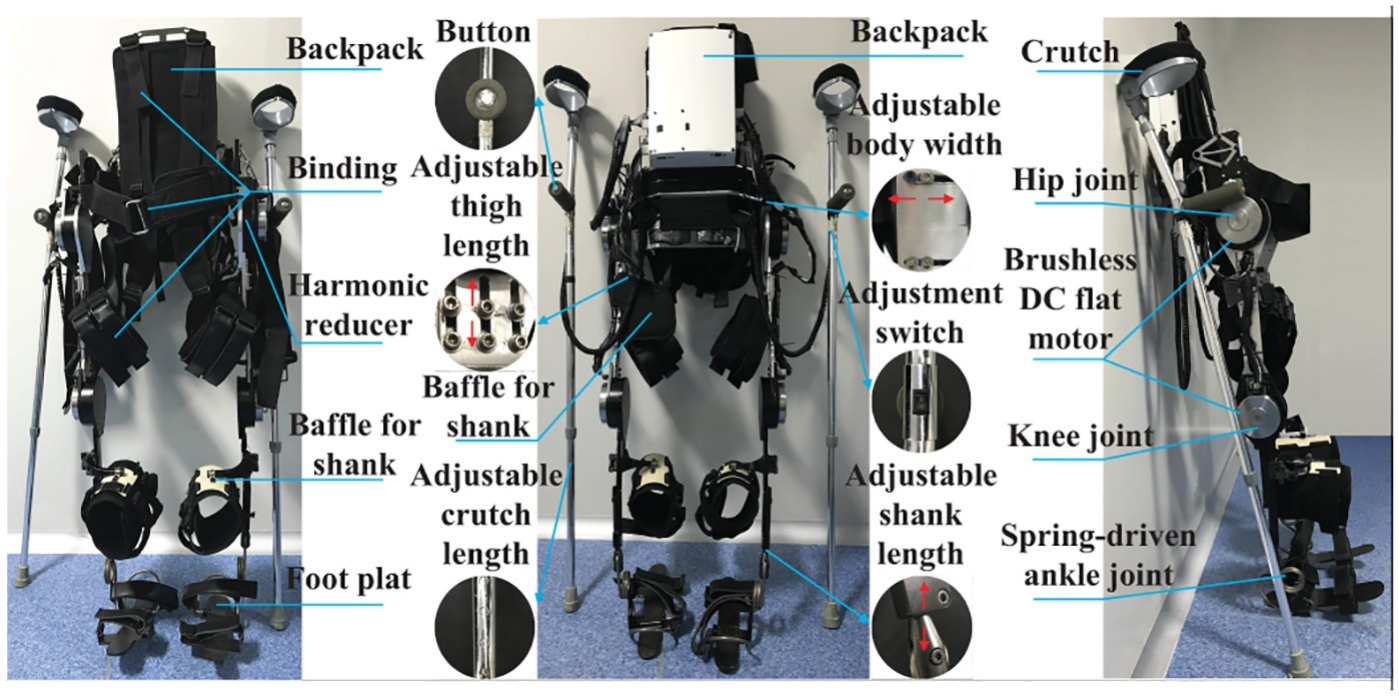
SIAT mechanical structure.

The SIAT exoskeleton weight is 15kg. The main drive is hip and knee joints. There are four degrees of freedom, which can help the hip and knee joints in the sagittal plane. Furthermore, the ankle joint is equipped with a spring mechanism to ensure the maximum contact area between the sole and the ground, increasing stability, as illustrated in Fig 2. Exoskeleton is suitable for people between 165 and 185cm in height. The mechanical connecting rod of the thigh, calf, and waist of the exoskeleton can be adjusted within a certain length range.

**Fig 3:**
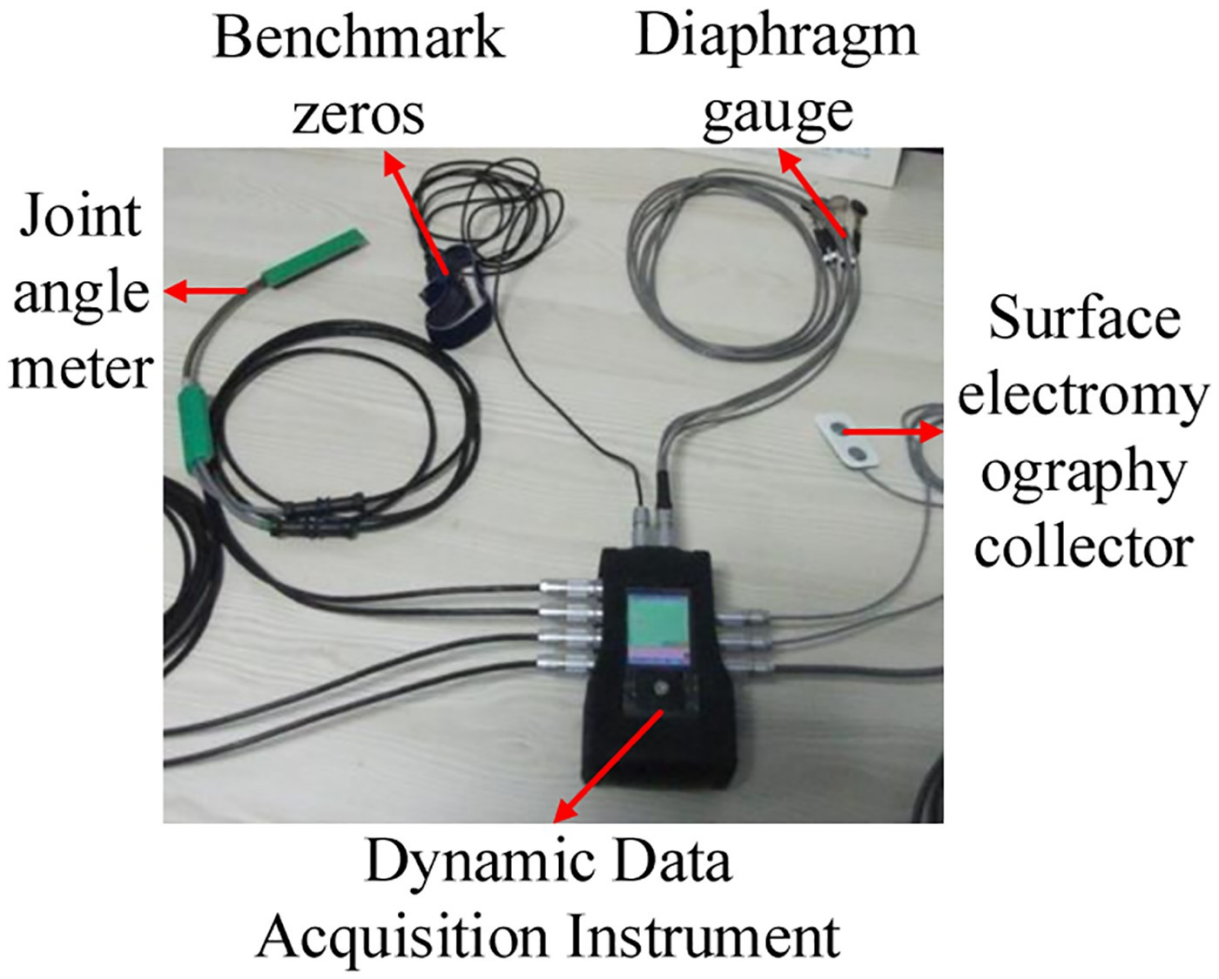
Biometrics sEMG acquisition equipment.

In this work, we investigate the realization of human motion intention prediction function through sEMG signals to enhance the human-computer interaction experience of exoskeleton robots. The sEMG signals acquisition device is a biometrics portable sEMG signals acquisition system, which is presented in Fig 3.

**Fig 4:**
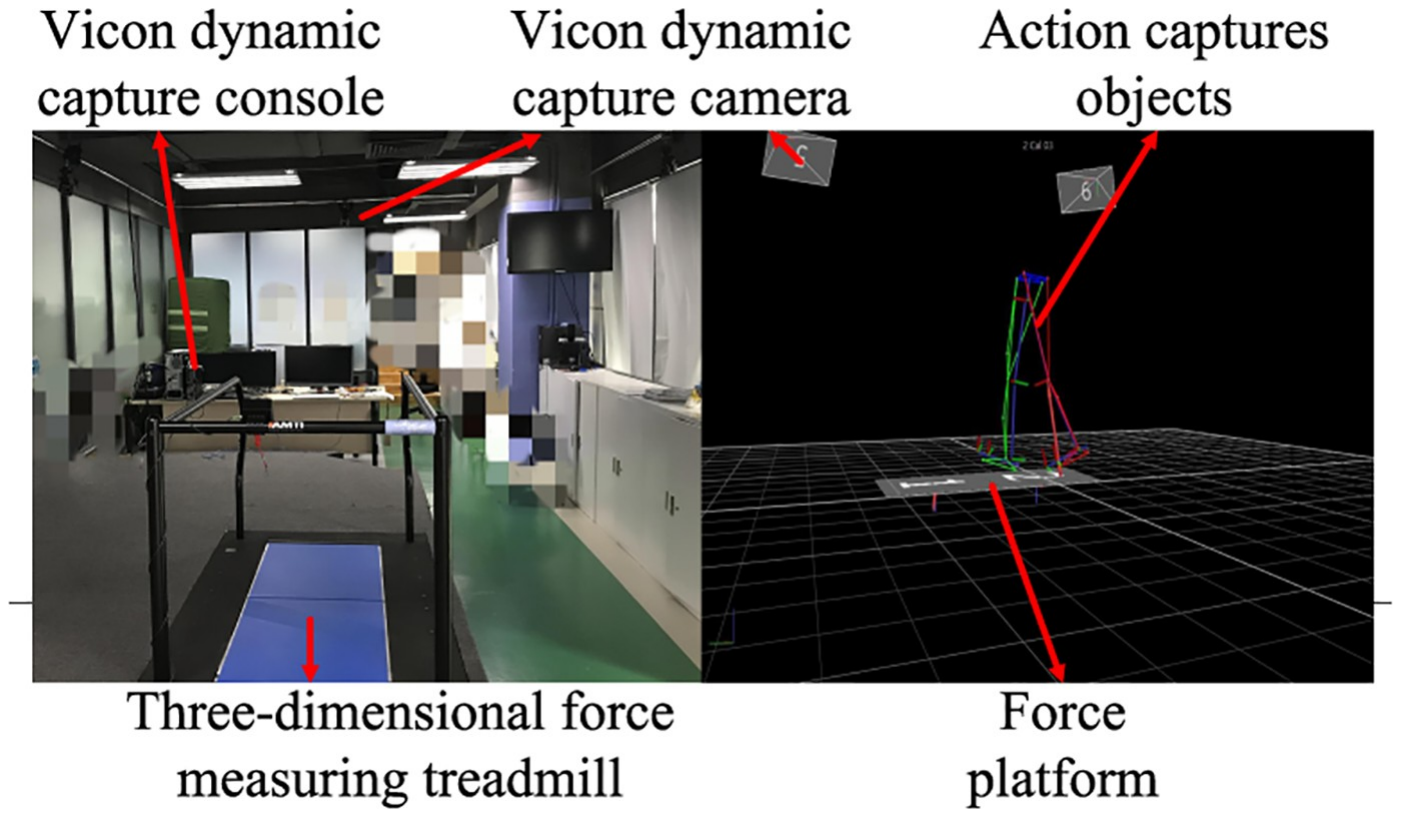
Experimental environment.

Vicon-Nexus (VN) can be used for real-time online or off-line motion capture and analysis. The working principle is based on a reflective capture system, which requires reflective balls on the wearer. By following VN’s control software processing, the 3D coordinates and the trajectory of each reflective ball can be obtained. In this study, six cameras were used to capture the motion as illustrated in Fig 4.

## Motion intention recognition and gait design analysis

Setting different gaits for different motion intentions is an important parameter of human-computer interaction. Support vector machine (SVM) is the only motion algorithm currently capable of decoding the intention signal in real time, and controlling exoskeleton robots on the master computer [28].

### Motion intention recognition

SVM is a linear classifier that minimizes the empirical error and maximizes the inter-class interval in the feature space. Its learning strategy is to maximize the class interval and thus transform the original two-class problem into a convex quadratic programming problem to solve.

Given a dataset composed of *m* pairs of data. *D* = {(*x*_1_, *y*_1_), (*x*_2_, *y*_2_), …, (*x*_*m*_, *y*_*m*_)} and label *y*_*i*_ ∈ (*- 1*, *+ 1*). A boundary can be constructed as:

$$\begin{array}{c} f(x)=w^{T}x+b=0 \end{array}$$ (1)

where *w* and *b* is the parameters to be defined from the optimization problem.

$$\begin{array}{c} \underset{w,b}{\text{min}}(\frac{1}{2}w^{T}w+C\sum_{i=1}^{m}\xi_{i})=0 \end{array}$$ (2)

where constant *C* is the regularization coefficient that controls the trade-offs between structural and empirical risks of the model. To solve the problem of overfitting which may occur in the process of SVM use, slack variable *ξ* is introduced, and the constraint condition of the SVM formula is changed to:

$$\begin{array}{c} s.t.\xi_{i}\geq 0 \end{array}$$ (3)

$$\begin{array}{c} y_{i}(w^{T}x_{i}+b)\geq 1-\xi_{i},i=1,2,\ldots ,m \end{array}$$ (4)

The SVM objective function also needs to be modified accordingly after introducing the relaxation variable *ξ*. Add the sum of squares of the upper relaxation variables and find the minimum to achieve a balance. Avoid over-merging to ensure certain stability.

**Fig 5:**
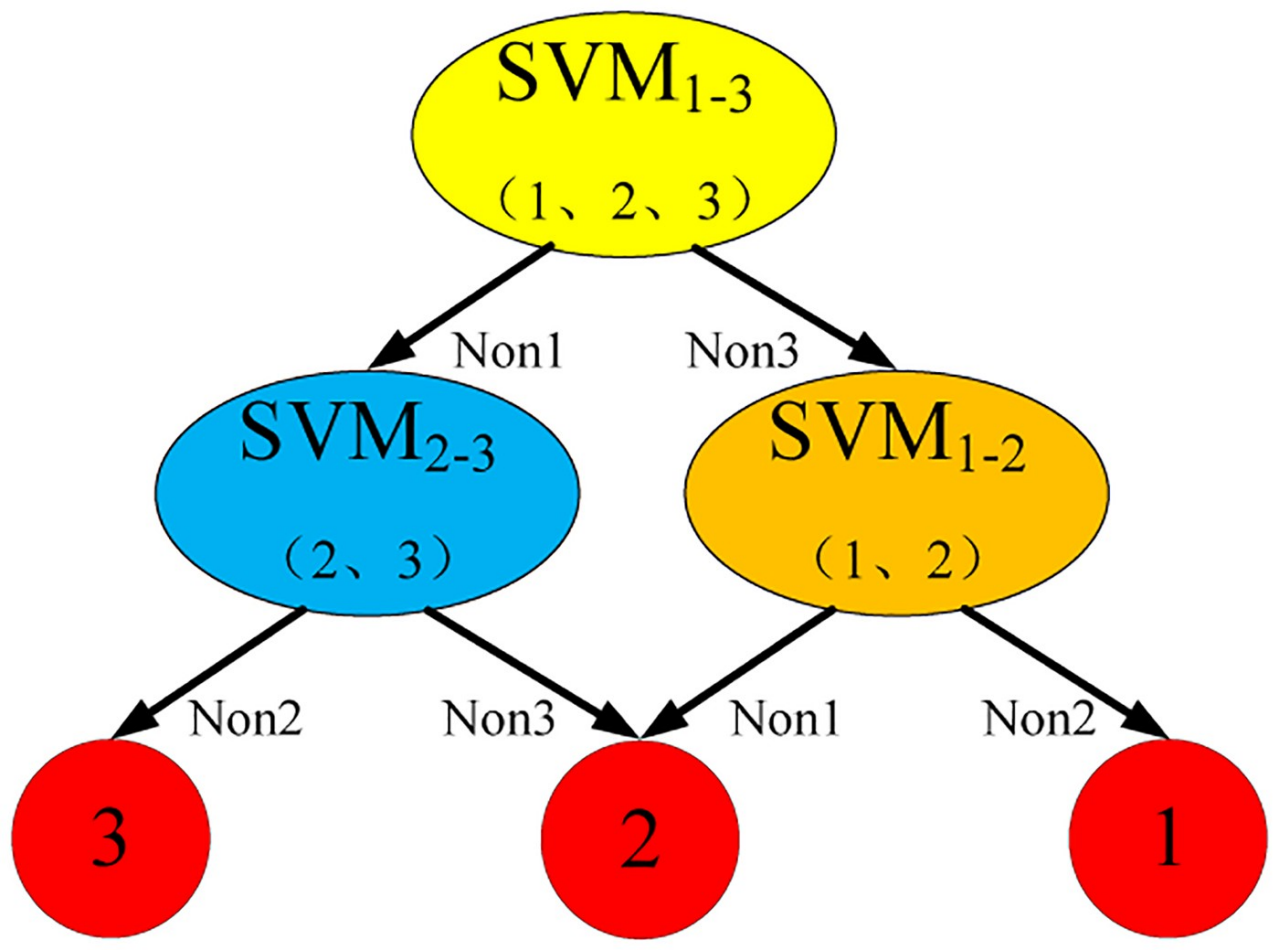
SVM intention-based classifications.

For the prediction of motion intention of multi-classification, SVM usually adopts two strategies to achieve multi-classification: one-to-many and one-to-one. A one-to-many strategy is to divide a class of samples into one class in turn and the rest into another class by training. *N* categories construct N SVM. In a one-on-one strategy, by training a SVM between any two classes of samples, the *N* categories need to design *N* × (*N* − 1)/2 SVM classifiers. For a class of unknown samples, the voting method is used to determine the sample as the largest number of votes. In this paper, a one-to-one strategy is used to construct an SVM classifier for motion intentional determination, as displayed in Fig 5.

### Gait analysis

**Fig 6:**
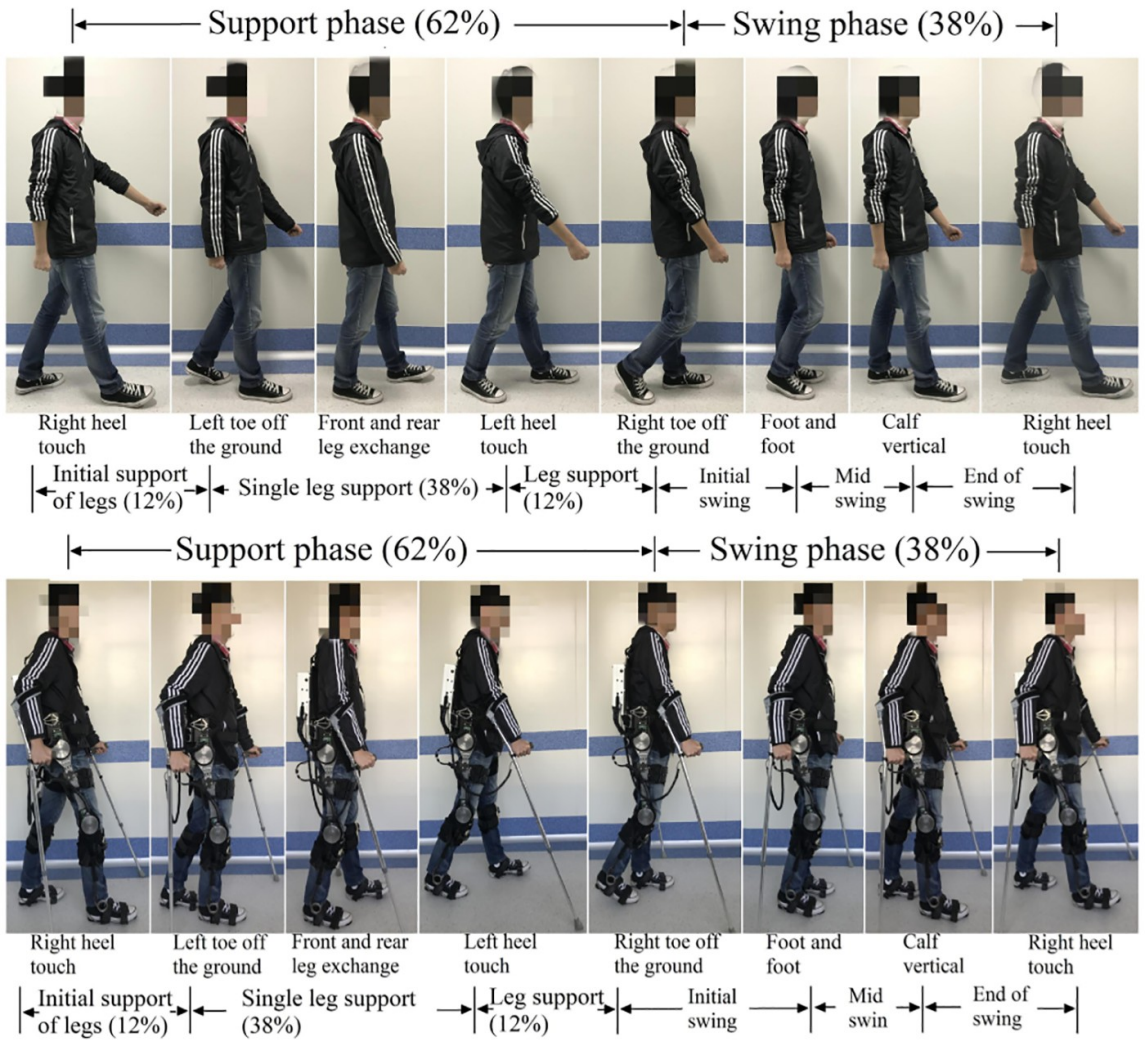
Walking phase.

By dividing the walking process of normal people’s walking cycle: 38% of the state is supported by one leg; 24% of the state is supported by the other leg; the remaining 38% of the state is half supported by both legs. As showing in Fig 6.

**Fig 7:**
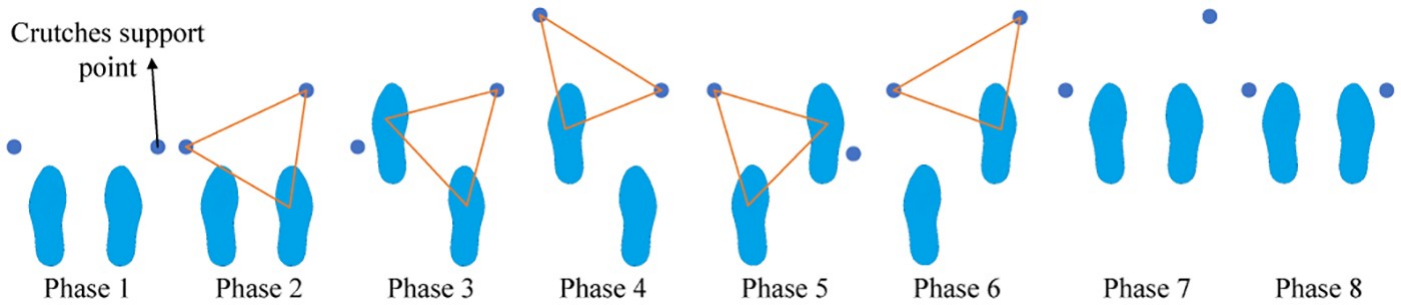
Motion phase structure diagram of exoskeleton robot.

A complete walking gait starts with the left leg to provide support; then both legs to provide support; and finally, the right leg to provide support as presented in Fig 6. Wearing exoskeletons and walking with crutches, a complete gait cycle can be divided into phases as displayed in Fig 7:

1. Move the right crutches and lift the left leg for the first step;
2. Move the left crutches and lift the right leg for a second step;
3. Move the right crutches and take the left foot;
4. The right crutch moves back to the initial position.

In this paper, by analyzing the motion data collected by VN equipment, we investigate the gait planning of exoskeleton wearers walking on horizontal ground, where the motion of the driving joints is constrained in the sagittal plane. The connecting rod model is used to approximate the body’s trunk and legs. The knee joint and the hip joint are the rotating axes of the connecting rod. During walking on the horizontal ground, the motion trajectories of the hip, knee, and ankle in the sagittal plane are periodic curves. When planning for the gait trajectory of the exoskeleton, we can use a sine wave and triangular wave to approximate the motion trajectory of the joint. When the trajectory of the joint is determined, the joint angle can be solved by using the link constraint. Finally, hip and knee joint cooperative control is achieved.

**Fig 8:**
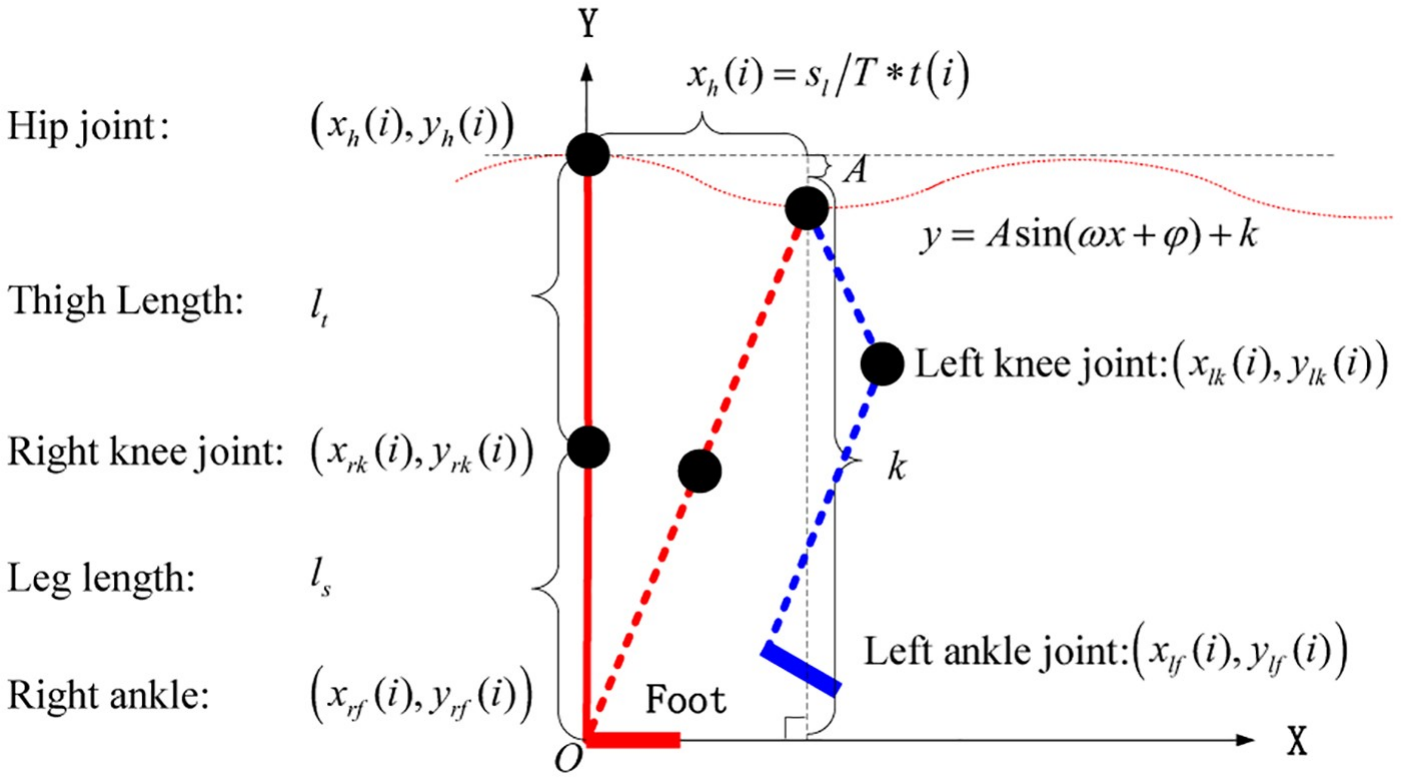
Connecting rod structure model.

In this work, a sine wave *y* = *A* sin(*ωx* + *φ*)+ *k* is used to plan the gait trajectory. The gait is divided into three stages: starting, walking, and closing. Only the model for the initial builds is listed here, as illustrated in Fig 8.

The starting stage sets the thigh length *l*_*t*_ (thigh), the leg length *l*_*s*_ (shank), the step height *s*_*h*_ (high), the step size *s*_*l*_, the period T, the sampling time *t*(*i*), the *i* representing a certain moment, the right-foot coordinates *x*_*rf*_(*i*) and *y*_*rf*_(*i*), and the initial time *i* = 0. The horizontal coordinate of the hip is *x*_*h*_(*i*) = *s*_*l*_/*T* * *t*(*i*), the ordinate is *y*_*h*_(*i*), amplitude is $A=l_{t}+l_{s}-\sqrt{{\left(l_{t}+l_{s}\right)}^{2}-{\left(s_{l}/2\right)}^{2}}$, constant is $k=(l_{t}+l_{s}+\sqrt{{\left(l_{t}+l_{s}\right)}^{2}-{\left(s_{l}/2\right)}^{2}})/2$, angular frequency is *ω* = 2*π*/*s*_*l*_, and initial phase is *φ* = π/2.

In the same way, we can find the left foot horizontal coordinate *x*_*lf*_(*i*) = 2_*sl*_/*T* * *t*(*i*). The left foot ordinates *y*_*lf*_(*i*) where amplitude *A* = *s*_*h*_/2. Constant *k* = *s*_*h*_/2, angular frequency *ω* = 2*π*/*s*_*l*_. Initial phase *φ* = *π*/2. The left knee *x*_*lk*_ and *y*_*lk*_ are calculated as follows.

$${\left(x_{lk}-x_{lf}\left(i\right)\right)}^{2}+{\left(y_{lk}-y_{lf}\left(i\right)\right)}^{2}=l_{s}^{2}$$ (5)

$$\begin{array}{c} {\left(x_{lk}-x_{h}(i)\right)}^{2}+{\left(y_{lk}-y_{h}(i)\right)}^{2}=l_{t}^{2} \end{array}$$ (6)

The direction of motion is *x* axis positive, and the left knee value takes the maximum value of *x*_*lk*_ and its corresponding *y*_*lk*_.

When *x*_*lf*_(*i*) = *x*_*h*_(*i*), we have *y*_*lk*_ = 0.5*y*_*h*_(*i*). In this time *x*_*lk*_(*i*) = (*x*_*h*_(*i*) + *x*_*lf*_(*i*))/2.

When *x*_*lf*_(*i*) = *x*_*h*_(*i*), we have *y*_*lk*_ ≠ 0.5*y*_*h*_(*i*). In this time *x*_*lk*_(*i*) = *x*_*lk*_(*i* − 1).

In the same way, the right knee position value takes the maximum value of *x*_*rk*_ and it’s corresponding *y*_*rk*_.

When *x*_*rf*_(*i*) = *x*_*h*_(*i*), we have *y*_*rk*_ = 0.5*y*_*h*_(*i*). In this time *x*_*rk*_(*i*) = (*x*_*h*_(*i*) + *x*_*rf*_(*i*))/2.

When *x*_*rf*_(*i*) = *x*_*h*_(*i*), we have *y*_*rk*_(*i*) ≠ 0.5*y*_*h*_(*i*). In this time *x*_*rk*_(*i*) = *x*_*rk*_(*i* − 1).

The angle of the reverse ch joint is:

Left hip angle:
  $$\begin{array}{c} ahl\left(i\right)=\text{arctan}(\frac{x_{lk}\left(i\right)-x_{h}\left(i\right)}{y_{h}\left(i\right)-y_{lk}\left(i\right)}) \end{array}$$ (7)
Right hip angle:
  $$\begin{array}{c} ahr\left(i\right)=\text{arctan}(\frac{x_{rk}\left(i\right)-x_{h}\left(i\right)}{y_{h}\left(i\right)-y_{rk}\left(i\right)}) \end{array}$$ (8)
Right knee angle:
  $$\begin{array}{c} akr\left(i\right)=\text{arctan}(\frac{x_{rf}\left(i\right)-x_{rk}\left(i\right)}{y_{rk}\left(i\right)-y_{rf}\left(i\right)}) \end{array}$$ (9)
Left knee angle:
  $$\begin{array}{c} akl\left(i\right)=\text{arctan}(\frac{x_{lf}\left(i\right)-x_{lk}\left(i\right)}{y_{lk}\left(i\right)-y_{lf}\left(i\right)}) \end{array}$$ (10)

**Fig 9:**
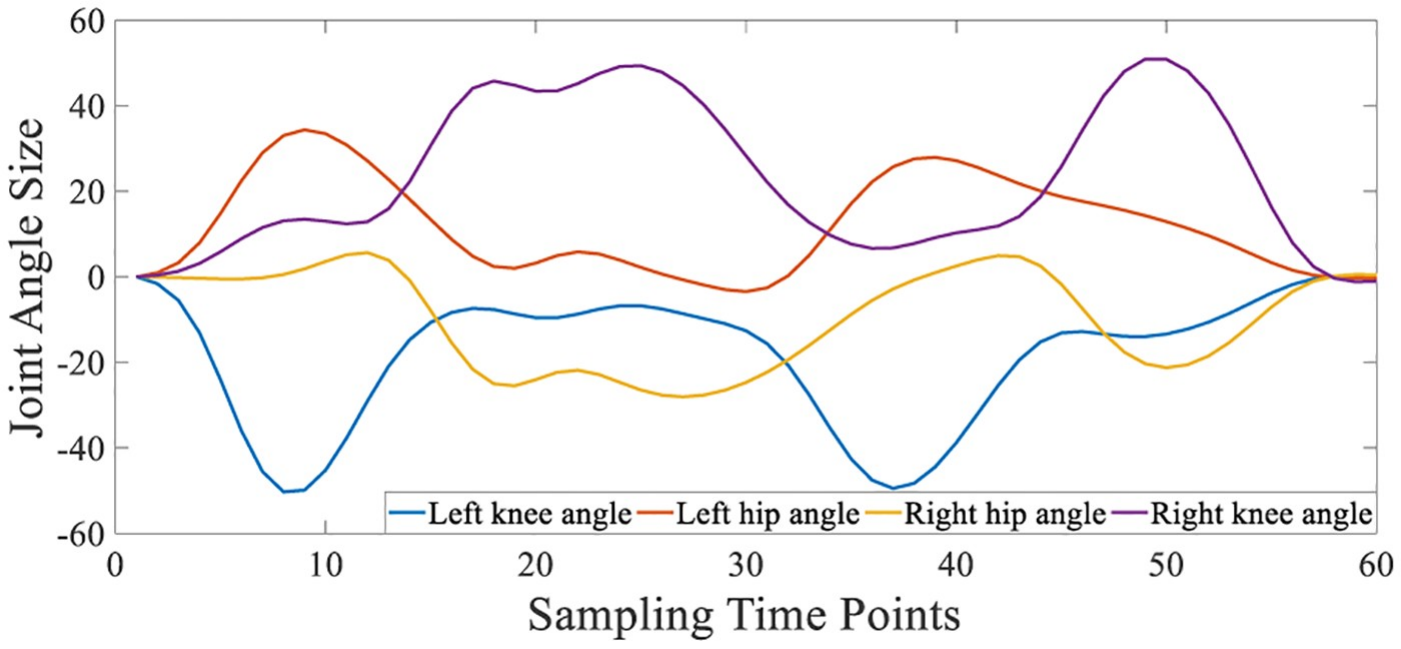
Four-degree-of-freedom joint angle.

The four-degree-of-freedom knee and hip joint angles are presented in Fig 9.

## Intention prediction and gait design

Planning and design of gait under different motion intentions will be presented in the paper. First, human motion intention is identified by decoding the original sEMG signal. Second, different motion gaits are established by modeling the exoskeleton motion state. Then the stability of the established gait is analyzed through the support structure and the correlation of joint angle and plantar pressure. Finally, Visual Studio is used to the exoskeleton robot control program through the control method of position, speed, and time. By listening for different signal coding under different intentions in real time, to realize switching different gaits according to different intentions. All subjects participated in this work voluntarily signed an informed consent form approved by the Medical Ethics Committee of Shenzhen Institutes of Advanced Technology ((SIAT)-IRB-170315-H0142).

### Movement intention

In this paper, three kinds of motion intentions are predicted and identified, which are: small step walking, stride walking, and stop. During wearer’s exoskeleton training, the wearer is trained to pass specific hand movements to reflect the corresponding motion intention. While collecting sEMG signals of specific muscles on the arm of feature coding, and the three motion intentions correspond to specific hand movements (as shown in Table 1) as follows:

1. Turn the wrist to represent a small walk;
2. To stand still means to stop walking;
3. A clenched fist represents a stride.

**Table 1:** Intended to identify action.

Movement intention	Small walk	Stop	Big strides
Category label	1	0	-1
Trigger action	Turn your wrists	Plant oneself	Grip

The training support vector machine (SVM) is used to classify the acquired sEMG feature vectors for motion intention. To realize the prediction of the wearer’s motion intention, and to achieve the purpose of gait switching of exoskeleton robots.

The sEMG signals acquisition sensor needs to keep close to the skin surface of the subjects during the signal acquisition process, considering the following two factors:

1. Signal acquisition sensors cannot interfere with other movements of exoskeleton wearers.
2. The sEMG signal is stable and reproducible.

**Fig 10:**
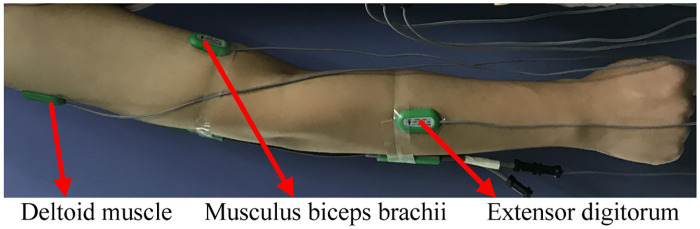
sEMG collection location.

So in this paper, we collected sEMG signals of three muscles of deltoid, biceps, and extensor (as displayed in Fig 10) for exercise intention prediction experiments.

**Fig 11:**
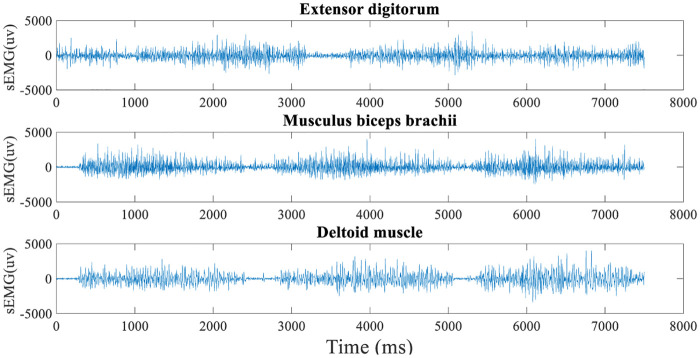
Primitive sEMG signal.

The sEMG signals acquisition frequency is 1000Hz. Subjects did the corresponding hand movements 10 times continuously under each exercise intention; a total of 45000 data points were collected. The original sEMG signal has a voltage range of 0 ∼ 1mV, as displayed in Fig 11. The trapping treatment of 50Hz eliminates power frequency interference. The infinite unit impulse response digital filter (IIR) performs bandwidth of 10 ∼ 500Hz bandpass filter. After signal preprocessing, feature extraction is carried out.

For sEMG signals, the commonly used feature extraction methods can be classified into three categories: time-domain analysis, frequency domain analysis, and time-frequency domain analysis. The root-mean-square (RMS) feature in the time-domain analysis can reflect the characteristics of sEMG signal amplitude with time, which is directly related to the electrical power of sEMG signals, and therefore has good real-time performance with a small delay. RMS gives the maximum likelihood estimation of amplitude in a constant force and non-fatiguing contraction while modeling a signal as a Gaussian random process [29].

**Fig 12:**
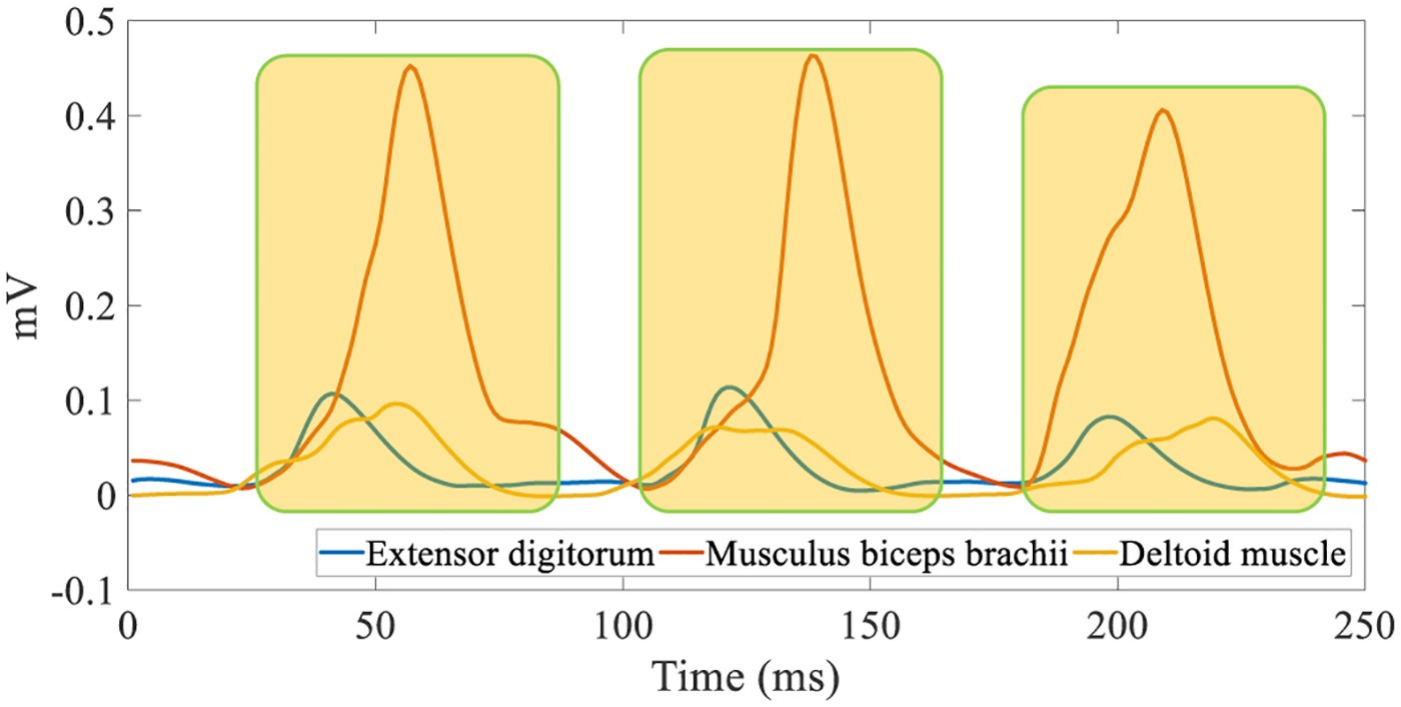
Feature vector extraction.

In this paper, RMS is utilized to extract the features of the sEMG signals. The sliding window scale of RMS is set to 20 and the moving step size of the window is set to 10. RMS features of the extensor, biceps, and deltoids can be obtained by the RMS feature extraction after the aforementioned pre-processing filter. The post-processing of the low pass filter is illustrated in Fig 12.

**Fig 13:**
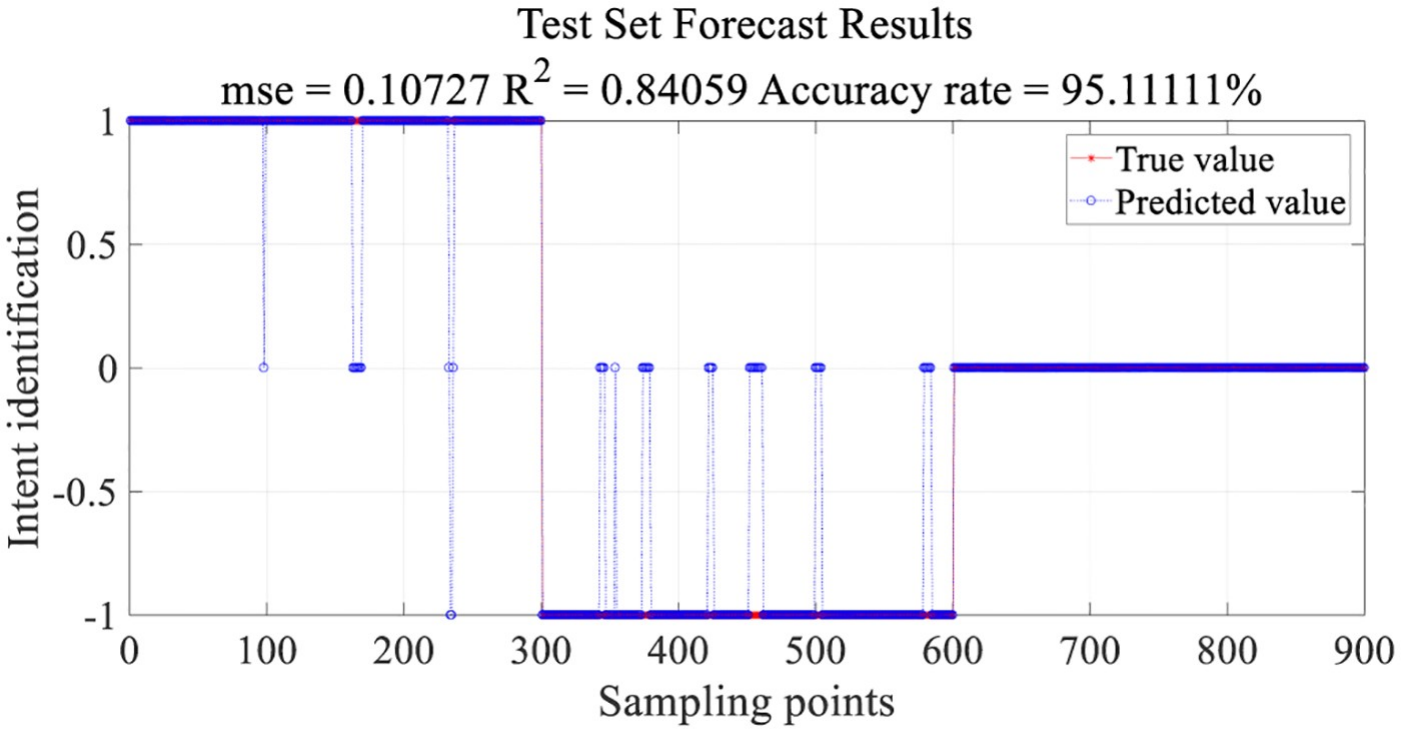
Test set forecast results.

Using libsvm to achieve multi-classification in MatLab, the kernel function is a Gaussian radial basis function kernel (RBF). The RMS feature of the three muscles at the same time can constitute a three-dimensional feature vector. The class label of the feature vector is given by the hand action at the corresponding moment. After feature extraction, a total of 4500 groups of feature vectors with class labels were obtained. Each action contains 1500 data samples. Distribute the whole data set as the training set: test set being at 80%:20%; find the best penalty coefficient *c* and the amplitude-width parameter *g* of *RBF* in the interval of [−10 − 10] per interval by cross-validation. The test set prediction results are obtained by inverse normalization. To reduce the number of error prediction results, the eigenvector of the predicted value belonging to the interval [−0.5 − 0.5] is judged as category 0. The eigenvector of the predicted value being greater than 0.5 is determined as category 1. The eigenvector of the predicted value being less than −0.5 is determined as category -1. Through the above processing, the test results for the test set are presented in Fig 13.

**Fig 14:**
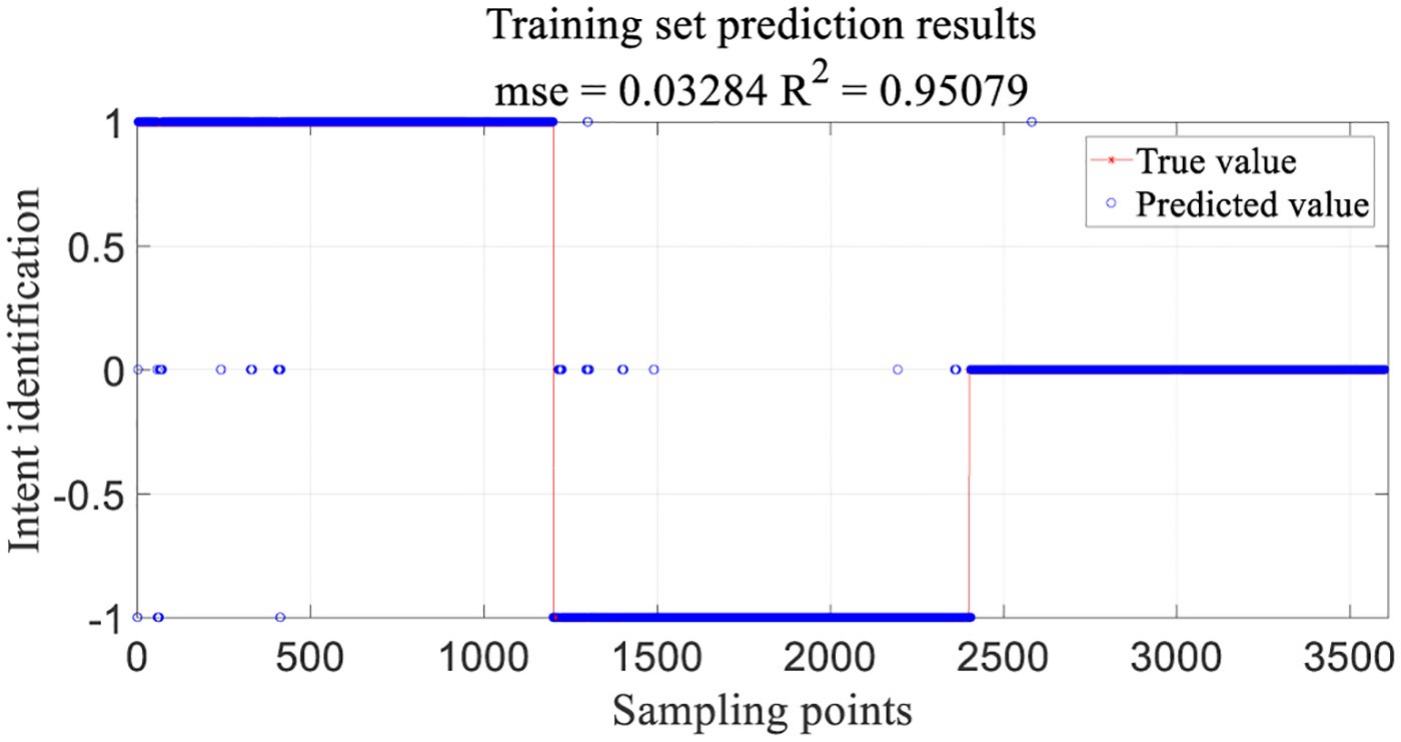
Training set identification results.

1, 0, -1 are the category labels for the three motion intentions of walking, stopping to walk, and striding respectively. The training set identifies the results, as displayed in Fig 14. * represents the true category label and o represents the predicted results.

The reliability of the intention recognition rate is verified by cross-validation. Comparing the prediction results after adjusting the fold number, when the folder number reaches 9 Fold, the SVM classifier has the highest accuracy of intention recognition on the test set, as shown in Table 2.

**Table 2:** Intentional recognition rate.

**F**old	5fold	7fold	9fold	12fold
Discrimination	95.12%	96.00%	96.68%	96.65%

Because the characteristics of different wearers’ movements are different, it is difficult to accurately classify the wearers’ intentions when the intention data are not in the database by the intention classifier trained by the data set. In order to verify the adaptability, we randomly selected 4 volunteer test intention data to test. To compare the effects of different predictors, we define the evaluation criteria as:

$$\begin{array}{c} MSE=100\times (\frac{1}{N}\times \sum_{i=1}^{N}({\left(y(i)-y_{p}(i)\right)}^{2})) \end{array}$$ (11)

Where *N* is the size of the test set, *y*(*i*) is the target value, and *y*_*p*_(*i*) is the predicted value. If the MSE is smaller, the error between the predicted and true values is smaller. If the MSE is larger, the error between the predicted value and the true value is larger. Hence the smaller the value of the MSE, the better the prediction effect.

Since SVM and BP neural network algorithms do not require iterative optimization, the predictor can be updated using current data after the prediction results. The MSEs of the two methods is shown in Table 3. The results show that the SVM algorithm has the smaller MSE value and the higher fitness.

**Table 3:** Mean square error of different algorithms.

Model	1	2	3	4	Average value
SVM	26.22	26.67	20.89	28.89	25.67
BP	30.22	29.78	24.89	28.89	28.44

### Gait design

According to the different motion intention establish the corresponding motion model, and planning gait suitable for a specific motion intention through a motion model.

#### Walking model

**Fig 15:**
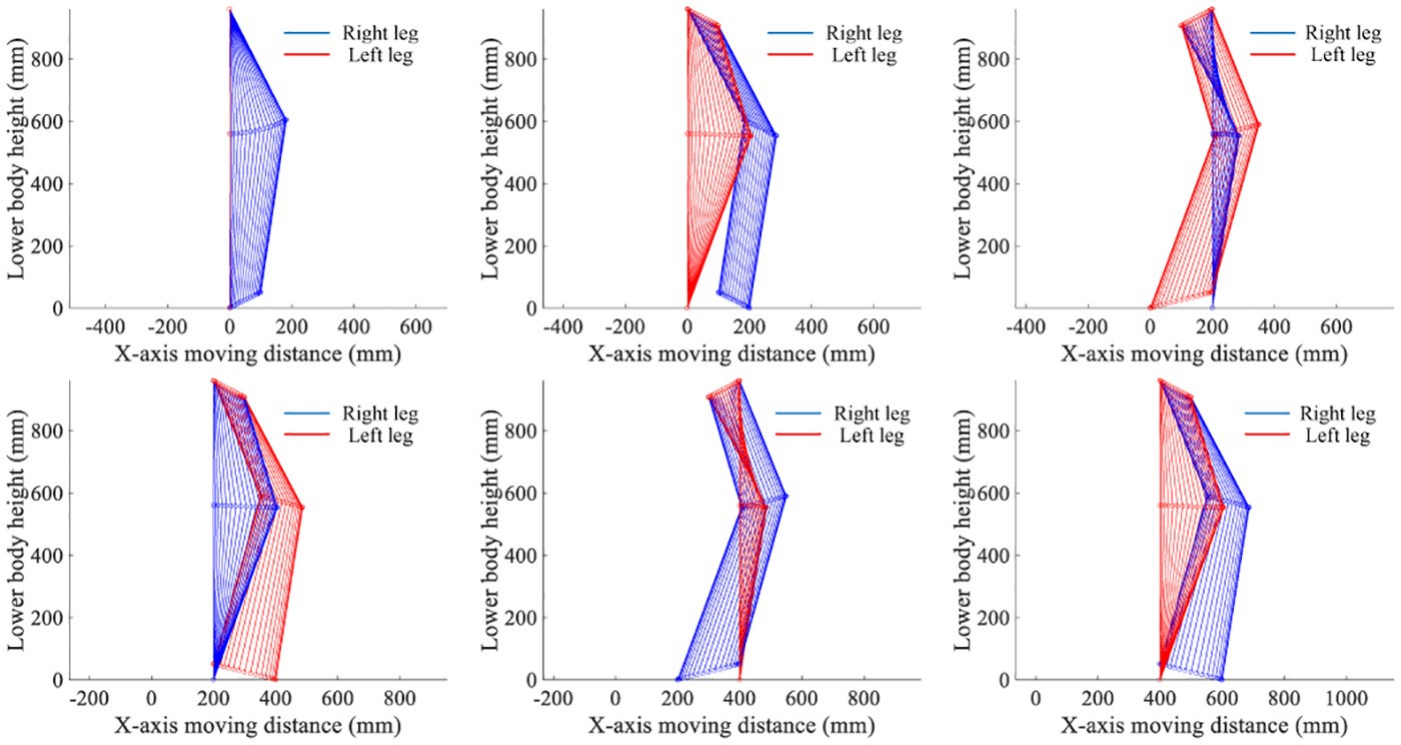
Motion model of exoskeleton robot.

Normal people mainly rely on the hip, knee, and ankle joints to achieve the goal of exercise walking. The ankle joint is used as the coordinate origin. The thigh and calf are regarded as two connected connecting rods. The motion model is planned as displayed in Fig 15.

To ensure the stability of the movement, 10 normal subjects were tested to lift the height of their legs while walking, as shown in Table 4.

**Table 4:** Height of foot lift during walking.

Height	165cm	175cm	179cm	183cm	185cm
1	11.1cm	11.8cm	11.2cm	11.7cm	11.5cm
2	11.2cm	11.7cm	11.6cm	11.6cm	11.5cm

**Fig 16:**
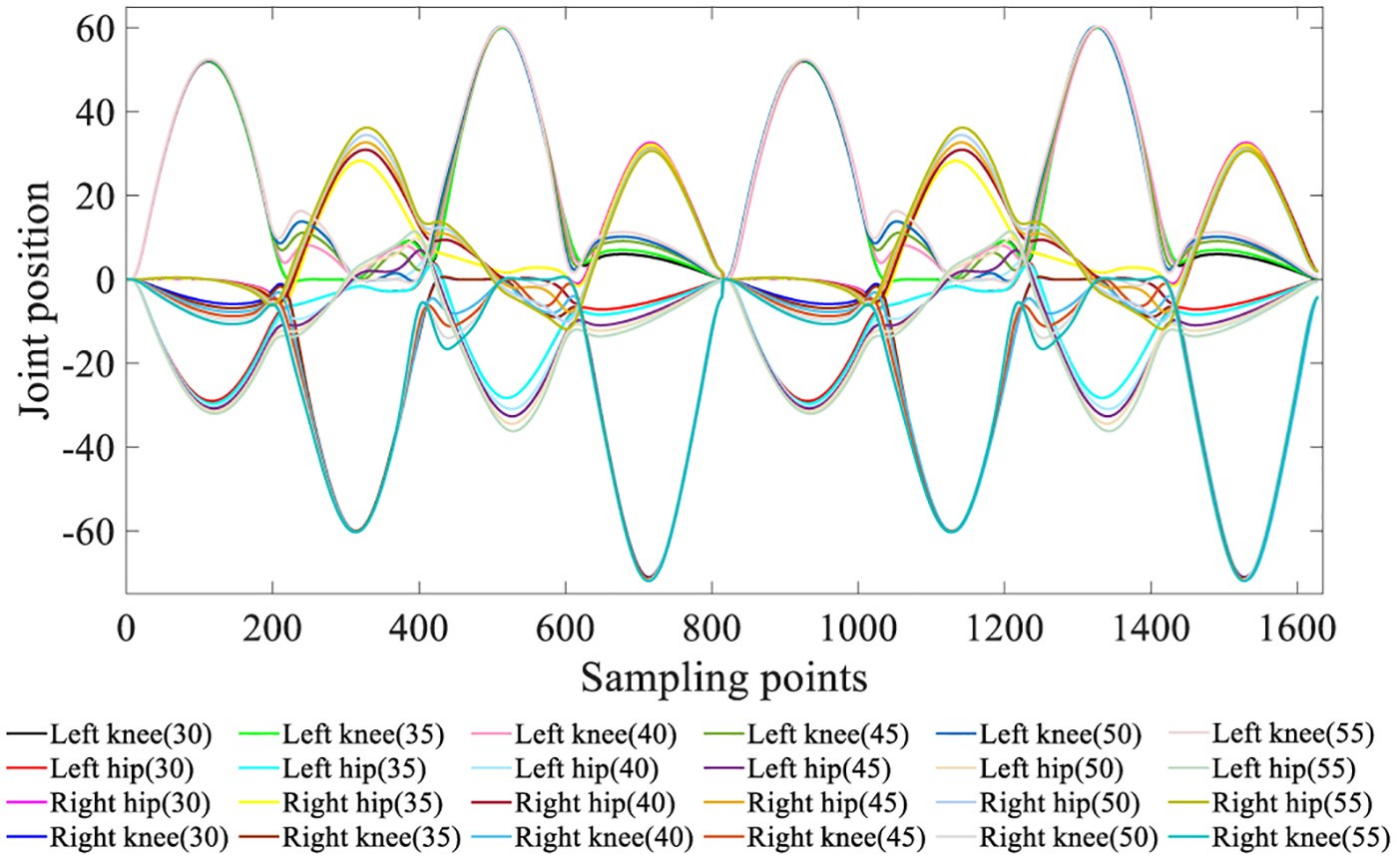
Planning gait trajectory.

Taking the intermediate value of 11.5cm as the lifting foot height, the joint motion trajectory map was planned according to the motion model, as displayed in Fig 16.

#### Gait stability validation

**Fig 17:**
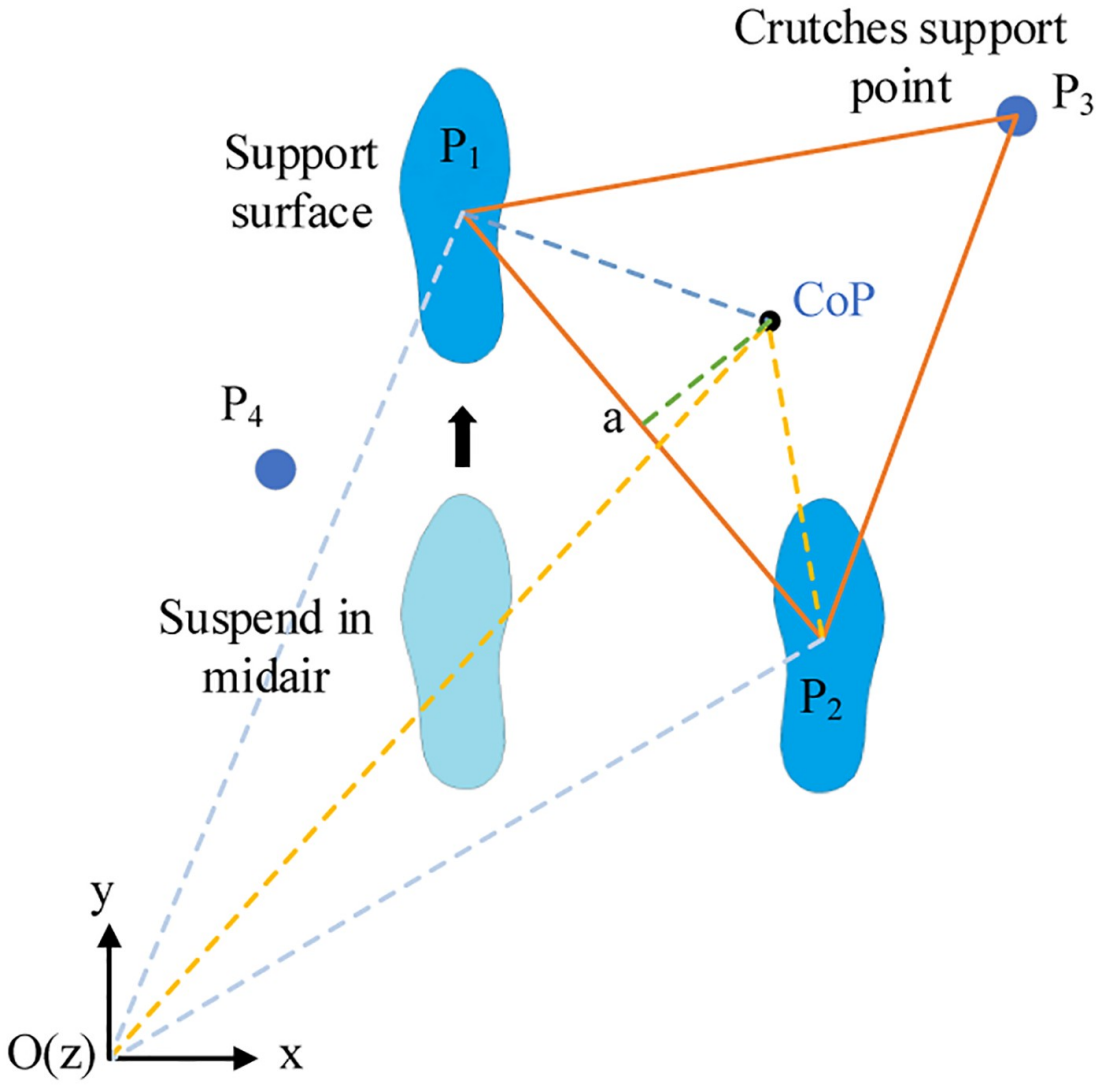
COP axis.

By marking the route and crutches support points position on the ground, exoskeleton wearers need to place the cane support point near the mark on the ground, as displayed in Fig 17. *α* is the shortest distance from the cop point to each side.

$$\begin{array}{c} \alpha_{ca}=d_{c⊥p_{1}p_{2}} \end{array}$$ (12)

$$\begin{array}{c} d_{c⊥p_{1}p_{2}}=|\vec{oc}-\vec{op_{2}}-(\vec{op_{1}}-\vec{op_{2}})\cdot (\vec{oc}-\vec{op_{2}})\vec{e}| \end{array}$$ (13)

**Fig 18:**
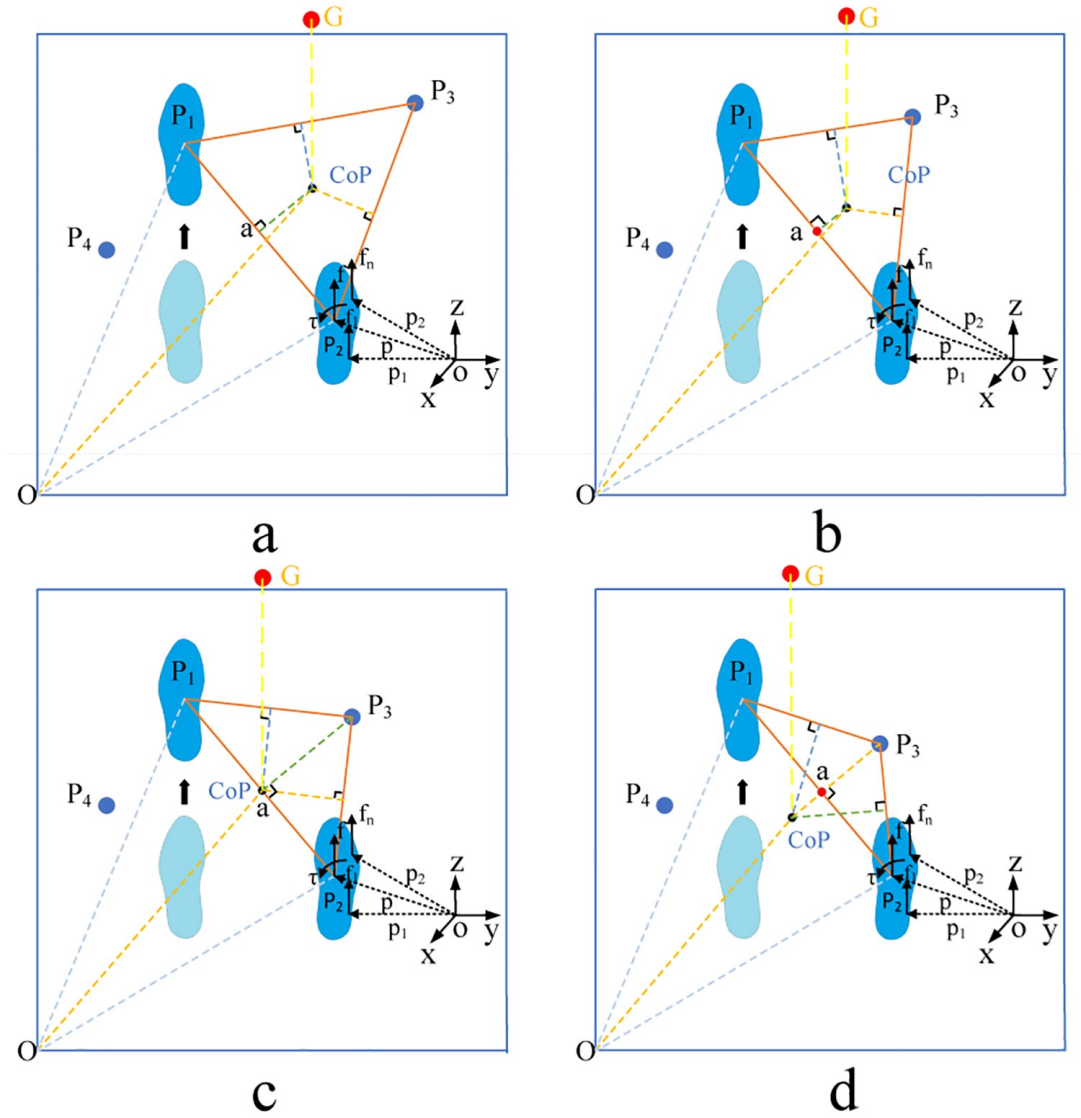
Kinematic support triangles for exoskeleton robot.

The supporting structure formed during the walking process is illustrated in Fig 18.

The position of the supporting point will affect the stability of the supporting structure. When the support structure is an acute triangle, the center of gravity will fall inside the triangle, forming a stable state; when the support structure is a right triangle, the center of gravity will fall on the oblique edge of the triangle, forming a critical stable state; when the support structure is an obtuse triangle, the center of gravity will fall outside the triangle, forming an unstable state.

Plantar pressure data (taking the average of the maximum supporting pressure) are collected through the VN 3D dynamometer treadmill equipment during the exoskeleton movement, shown in Table 5.

$$\begin{array}{c} r=\frac{\sum \left(x-\bar{y})(y-\bar{y}\right)}{\left(\sqrt{\sum_{i}^{n}{\left(x_{i}-\bar{x}\right)}^{2}})(\sqrt{\sum_{i}^{n}{\left(y_{i}-\bar{y}\right)}^{2}}\right)} \end{array}$$ (14)

Where *x* represents plantar pressure and *y* represents a joint angle. The average correlation coefficient of plantar pressure with joint angle *r* is shown in Table 6.

**Table 5:** Support point pressure at different steps.

Height	165cm	175cm	179cm	183cm	185cm	185cm
Half step	N/kg					
Left crus	15.31	15.40	15.42	15.54	14.46	15.66
Right crus	15.35	15.42	15.39	15.56	14.76	15.56
Stride	N/kg					
Left crus	16.47	16.66	17.45	17.23	16.32	17.66
Right crus	16.50	16.62	17.28	16.74	16.46	17.54

**Table 6:** The correlation coefficient between the angle of the joint and the pressure of the foot in different steps.

Parameter	30cm	35cm	40cm	45cm	50cm	55cm
r	0.872	0.915	0.931	0.895	0.864	0.813
r	0.743	0.825	0.860	0.853	0.761	0.759

The higher the correlation coefficient, the more stable the gait is [30]. From Table 5, we found that *r* > 0.7 illustrated the strong correlation and the planned gait tended to be stable.

**Fig 19:**
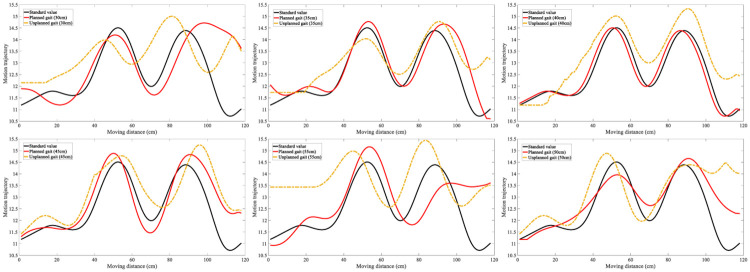
Trajectory of motion center of gravity change.

The gait center of gravity after filtering with steps of 30, 35, 40, 45, 50, and 55cm are plotted, as displayed in Fig 19. Each gait in each image was calibrated with standard values (collected by Vicon dynamic capture), planned gait (walking according to the planned gait), and unplanned gait (not walking according to the planned gait). Comparing the difference between the two gaits and the standard values in each group, we found that the planned gaits were more similar to the standard values, and the peaks and valleys of the center of gravity track were more similar in the stable walking.

### Real-time intent signal control

In order to be able to obtain the motion intention in real-time, the gait of the exoskeleton robot is changed by different motion intention. We need to build a multi-system fusion control system. In the lower limb control module of the exoskeleton robot, we refer to reference [31] to realize asymptotic stability analysis and state feedback control design of discrete-time switched nonlinear systems by the smoothing approximation technique. In terms of multi-system fusion synchronization with different parameters, we refer to reference [32] to ensure uniform synchronization within the specified error range. Visual Studio is used to establish exoskeleton Windows presentation foundation (WPF) control interface; establishing an SVM-based Matlab intention recognition program; IP address and port are used to establish a real-time communication connection with exoskeleton WPF control interface. Building a real-time communication connection between sEMG signals acquisition system and Matlab to make it run simultaneously.

**Fig 20:**
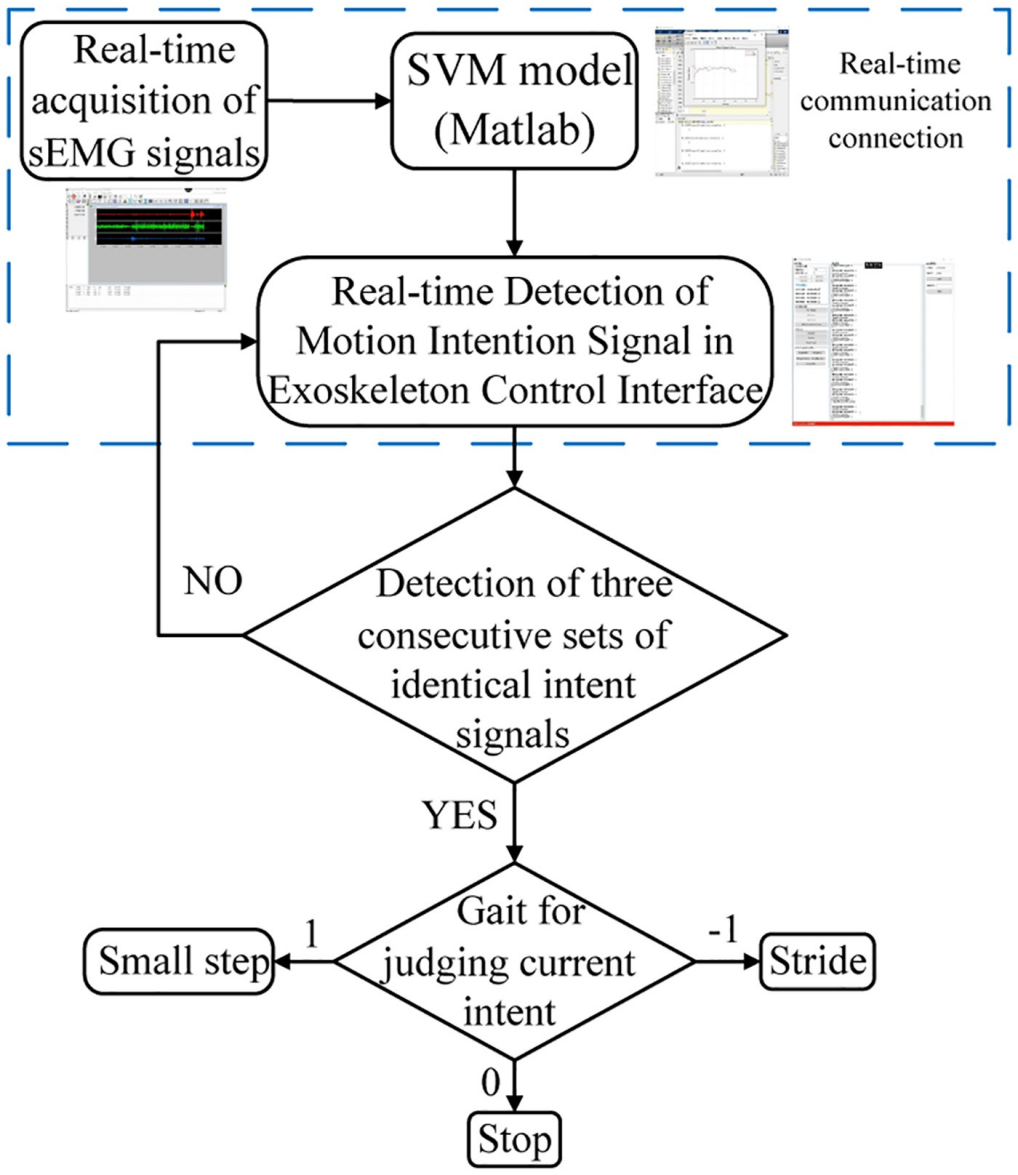
Real-time intent control gait block diagram.

sEMG real-time signal acquisition system sends the collected sEMG signal continuously to the SVM model established by Matlab and recognizes the human motion intention by decoding the sEMG signal. Different motion intention sets different coding. When the intention of small step walking is identified, the system sends the coding signal 1 to the background; for the stop motion intention, the system sends the coding signal 0 to the background; for the intention of stride walking, the system sends the coding signal -1 to the background. Exoskeleton control interface listens to the identified intention signal coding in real time, and runs the control program corresponding to the coding in the control interface to complete the gait switching. The ultimate realization through different intentions to switch the gait corresponding to the intention, the real-time intentional control block diagram is shown in Fig 20.

## Conclusion

The recognition of the specific action of the hand can be well realized by the SVM classifier and the collected sEMG signals, and then the recognition of the motion intention of the wearer of the exoskeleton can be realized. sEMG signal real-time acquisition system, intention recognition program, exoskeleton control program can be achieved at the same time. Exoskeleton wearers can control the gait switches in real time according to the motion intention. Avoiding complicated control methods can effectively enhance the human-computer interaction experience of the exoskeleton. It improves the stability of the control system and reduces the error of system synchronization. Meanwhile, the gait model planned in this paper can construct a stable exoskeleton gait trajectory and the control trajectory of the driving unit to the motor according to the two parameters of step height and step size. Stable gait can help patients to speed up the recovery of leg muscle vitality rehabilitation training.

In future work, we plan to fuse the sEMG signals with the measurement signal of the inertial device using multi-mode sensor signal fusion to achieve more accurate human intention recognition. We will realize the real-time control function of the exoskeleton according to the wearer’s motion intention, so as to get a better wearable experience.
